# Identifying Cortical Molecular Biomarkers Potentially Associated with Learning in Mice Using Artificial Intelligence

**DOI:** 10.3390/ijms26146878

**Published:** 2025-07-17

**Authors:** Xiyao Huang, Carson Gauthier, Derek Berger, Hao Cai, Jacob Levman

**Affiliations:** 1 Department of Computer Science, St. Francis Xavier University, Antigonish, NS B2G 2W5, Canadax2021cqq@stfx.ca (C.G.);; 2 Nova Scotia Health Authority, Halifax, NS B3H 1V8, Canada

**Keywords:** learning, proteins, mice, machine learning, artificial intelligence, feature selection, pruning, apoptosis

## Abstract

In this study, we identify cortical molecular biomarkers potentially associated with learning in mice using artificial intelligence (AI), inclusive of established and novel feature selection combined with supervised learning technologies. We applied multiple machine learning (ML) algorithms, using public domain ML software, to a public domain dataset, in order to support reproducible findings. We developed technologies tasked with predicting whether a given mouse was shocked to learn, based on protein expression levels extracted from their cortices. Results indicate that it is possible to predict whether a mouse has been shocked to learn or not based only on the following cortical molecular biomarkers: brain-derived neurotrophic factor (BDNF), NR2A subunit of N-methyl-D-aspartate receptor, B-cell lymphoma 2 (BCL2), histone H3 acetylation at lysine 18 (H3AcK18), protein kinase R-like endoplasmic reticulum kinase (pERK), and superoxide dismutase 1 (SOD1). These results were obtained with a novel redundancy-aware feature selection method. Five out of six protein expression biomarkers (BDNF, NR2A, H3AcK18, pERK, SOD1) identified have previously been associated with aspects of learning in the literature. Three of the proteins (BDNF, NR2A, and BCL2) have previously been associated with pruning, and one has previously been associated with apoptosis (BCL2), implying a potential connection between learning and both cortical pruning and apoptosis. The results imply that these six protein expression profiles (BDNF, NR2A, BCL2, H3AcK18, pERK, SOD1) are highly predictive of whether or not a mouse has been shocked to learn.

## 1. Introduction

How the brain implements learning is a fundamentally unsolved problem in science [1]. Although it is likely that learning occurs in the cerebral cortex, models of brain function and development that demonstrate how learning unfolds naturally are lacking. This study focuses on a dataset [2] that includes mice of eight types, half of which were subjected to a shock in an effort to force the mice to learn. All mice had molecular biomarker measurements of protein expression levels taken from their cerebral cortices. By creating feature selection (FS) and machine learning (ML) technologies, we can identify sets of feature measurements (cortical molecular biomarkers) that together are highly predictive of whether or not a given mouse was shocked to learn. This, in turn, may help the scientific community better understand underlying factors associated with learning.

The dataset relied upon in this study [2] includes not only mice that were and were not shocked to learn, but also mice with and without Down syndrome (Ts65Dn model), and with and without memantine treatment. Although the focus of this study is on whether or not the mice were shocked to learn (also referred to as contextual fear conditioning), it should be noted that Down syndrome (DS) is a common genetic cause of learning/memory deficits [3], and is caused by an extra copy of chromosome 21 [4]. As such, mice with DS may exhibit unique profiles of protein expression in response to learning as compared with non-DS mice. Protein genomic analyses provide a functional context for explaining genomic abnormalities and offer a new paradigm for understanding biology [5]. Thus, research utilizing ML with FS to identify specific proteins predictive of learning status may assist in improving our understanding of the natural brain processes that support the fundamentals of learning.

AI is widely used in the classification and analysis of proteins, where the application of ML technology has become relatively mature. For example, previous research has proposed the use of artificial neural networks to predict protein structure from amino acid sequences [6]. Additional research has focused on identifying biomarkers that distinguish Alzheimer’s disease, which is highly prevalent among individuals with DS, from other neurodegenerative illnesses [7], demonstrating that proteins and mRNA levels can be predictive of the development of the condition. The large amount of data being acquired in modern biology- and medicine-based research motivates the use of AI technology in this field [8], as ML models are particularly well suited to process and analyze extensive datasets.

### 1.1. Closely Related Work

A variety of studies have been conducted [4,9,10,11,12] focused on the same public domain dataset [2] that is being addressed in this study. During trials conducted on mice, the drug memantine’s administration resulted in improvements in learning in Ts65Dn mice (mice with DS) [4]. They observed that memantine administration does not immediately result in a normalization of protein levels; however, by the end of the repeated learning test, about half of the proteins achieved normalization of expression levels.

An additional analysis was performed with the use of the self-organizing map (SOM) [9], an unsupervised learning approach based on artificial neural network technology. The SOM method assisted researchers in reducing the large set of proteins to a subset potentially crucial to learning. Their analysis identified 12 features (see their Supplementary Figure S1, in [9]) with potential for discrimination between mice shocked to learn, and those not [9]. The identified protein expression biomarkers include DYRK1A, pGSK3B, pERK, CaNA, SOD1, pNUMB, ITSN1, IL1B, ubiquitin, PKCA, pPKCAB, and P38. Additionally, three studies exist that have taken a more standard supervised ML approach, creating AI technology that targets eight different classes present in the dataset (mice with and without being shocked to learn, with and without DS, and with and without memantine treatment). These approaches, while creating technology of potential interest, were not focused on identifying underlying factors that may be associated with learning specifically. Reported results include AI technology with 99.4% accuracy using a hyperparameter-tuned support vector machine [10], 100% accuracy using random forests [11], and 99.5% also using random forest models [12].

### 1.2. Hypothesis

We hypothesize that the use of open-source machine learning software, inclusive of extensive feature selection and supervised learning technologies, may produce helpful technology for identifying brain proteins potentially associated with learning.

## 2. Results

### 2.1. Predicting Whether a Mouse Was Shocked to Learn

Table 1 provides the results from our thorough analysis comparing a wide range of machine learning techniques, exhaustively combined with our supported feature selection methods, using 5-Fold cross-validation. Results indicate that our best-performing technologies were obtained with models based on either stochastic gradient descent (sgd) or logistic regression (lr), combined with our novel redundancy-aware feature selection method (wrap). The redundancy-aware feature selection method selected only six features, which are provided in Table 2. Note that higher accuracy score values in the table imply better predictive performance for that feature. Results indicate multiple models with high accuracies (100%) in predicting whether a mouse was shocked to learn based on protein expression levels of brain-derived neurotrophic factor (BDNF), NR2A subunit of N-methyl-D-aspartate (NMDA) receptor, B-cell lymphoma 2 (BCL2), histone H3 acetylation at lysine 18 (H3AcK18), protein kinase R-like endoplasmic reticulum kinase (pERK), and superoxide dismutase 1 (SOD1).

### 2.2. Results of the Alternative Approach to Detecting Potentially Learning-Linked Proteins

The original mouse experiments that produced the public domain dataset included memantine treatments, which were reported to help Down syndrome (DS) model mice recover their ability to learn [4], implying that DS mice were not learning appropriately without the therapeutic treatment. This motivated a run of our analysis whereby the group-of-interest includes all mice shocked to learn, except those from the DS model who did not receive memantine treatment, as this configuration could theoretically better differentiate between those mice that do and do not successfully learn. Results for 5-fold validation are provided in Table 3. Results indicate a reduction in predictive accuracy relative to the earlier experiment targeting the prediction of whether the mice were shocked to learn (from 100% to 87.7% for leading models). The leading performance was obtained with the embedded lgbm (embed_lgbm) feature selection method, which based its predictions on 15 protein expression features. However, our redundancy-aware feature selection method (wrap) combined with the random forest (rf) was competitive, demonstrating predictive accuracy (87.2%) roughly on par with leading findings, based on a reduced set of 12 features, which are provided in Table 4.

### 2.3. Visualization of Findings

**Figure 1:**
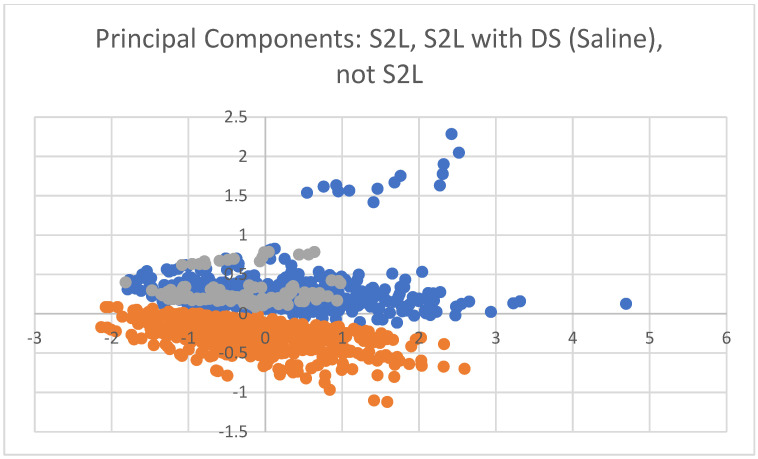
Two-dimensional principal components projection plot of six-dimensional data space created by our leading features in Table 2. Blue dots represent the shocked-to-learn (S2L) group, grey dots represent the Down syndrome group shocked to learn and given saline, and orange dots represent the not shocked-to-learn group. The x-axis is the first principal component; the y-axis is the second principal component.

A principal components projection plot was created to illustrate visually, a two-dimensional projection of the six-dimensional dataspace formed by the leading six features (see Table 2), and is provided in Figure 1. This projection captures 95.39% of the variance in the six-dimensional dataset. Results demonstrate a clear separation in this dataspace between mice that were shocked to learn and those that were not. Results also demonstrate overlap between DS mice shocked to learn and provided with saline (no treatment) and the rest of the mice that were shocked to learn.

## 3. Discussion

The results of this study provide strong evidence that key protein expression levels are predictive of learning behavior (in this case, shocked-to-learn status) in mice, as reflected by the performance of the leading models in Table 1. Indeed, Figure 1 confirms visually that the shocked to learn group is clearly distinct in this dataspace from the not shocked to learn group.

### 3.1. Protein Expression Potential Significance

The combination of the six identified protein expression biomarkers—brain-derived neurotrophic factor (BDNF), NR2A subunit of N-methyl-D-aspartate (NMDA) receptor, B-cell lymphoma 2 (BCL2), histone H3 acetylation at lysine 18 (H3AcK18), protein kinase R-like endoplasmic reticulum kinase (pERK), and superoxide dismutase 1 (SOD1)—are highly predictive of a mouse’s shocked to learn status, achieving 100% predictive accuracy with multiple learning machines (see Table 1).

Five of the six identified proteins have pre-existing links with learning in the scientific literature. Histone H3 acetylation at lysine 18 (H3AcK18) has previously been linked with learning, demonstrating dependencies on the intensity of training [13,14]. One might expect that being shocked to learn would constitute intense training, thus H3AcK18 expression levels might provide a strong biomarker predictive of shocked to learn status. Furthermore, reductions in expression or activity of protein kinase R-like endoplasmic reticulum kinase (pERK) have been associated with neuronal excitability and cognitive function and enhanced hippocampal-dependent learning, as well as memory [15]. Additionally, a mutant SOD1 mouse model exhibited a delay in learning and impaired long-term memory [16], implying a link between SOD1 and learning. It should also be noted that superoxide dismutase 1 (SOD1) is located on chromosome 21, for which three copies exist in trisomy 21 (Down syndrome—DS) model mice. As such, expression levels of SOD1 may assist our AI technologies in modeling learning status in the context of a population with variation in genetic state (DS vs. normal). BDNF is known to play “an important role in neuronal survival and growth, serves as a neurotransmitter modulator, and participates in neuronal plasticity, which is essential for learning and memory” [17]. NR2A are subunits of N-methyl-D-aspartate (NMDA) receptor complexes, which are known to contribute to controlling “neuronal plasticity associated with learning, memory and development” [18]. Memantine is also an NMDA receptor antagonist [4], and so NR2A expression may also be linked with modeling learning status in the context of a population with variation in treatment state (memantine vs. saline) by the AI.

B-cell lymphoma 2 (BCL2) is a protein that helps to control apoptosis, the process by which a cell undergoes programmed and controlled/directed death [19]. Although BCL2 has been linked with neural activity [20], a direct link between BCL2 and learning in the literature remains elusive. However, BCL2 expression has been linked with aCASP3 expression, which influences synaptic pruning [21,22]. Pruning is the process by which neural pathways are pruned/removed from the brain. Of the previously introduced five proteins included in our leading AI model, two also have clear links to pruning in the literature; it has been reported that BDNF is essential for activity-dependent pruning [23], and NMDA, of which NR2A is a subunit, has been reported to modulate the rate of pruning [24]. The link between BCL2 and learning is the least clear, with each of the other five proteins demonstrating previously known links to learning in the literature. BCL2 controls apoptosis [19], the process of programmed/directed cellular death. Thus, our findings imply that pruning and apoptosis may be associated with learning, as pruning- and apoptosis-linked proteins have been demonstrated to be predictive of learning (mouse learning status) with AI. Learning involves changes in brain function, thus it should be noted that pruning (signal pathway removal) and apoptosis (cell death/removal) are expected, in many situations, to cause changes in brain function, due to their effect on structural changes in the brain (i.e., it is unlikely for these structural brain changes to not be associated with functional changes). Thus, our findings imply that future research should consider the possibility that pruning and apoptosis are contributing processes to learning in the brain. Indeed, research in the literature has previously implicated apoptosis in learning tasks through adaptive models of artificial neural network activity [25].

In summary, five of the six protein expression features identified by our novel redundancy-aware feature selection method have the potential to be linked with aspects of learning based on literature findings alone. Our results from Table 1 indicate that the collection of these six protein expression levels (see Table 2) is predictive of whether a mouse was shocked to learn with 100% accuracy via multiple machine learning methods. Thus, these findings may imply that these particular proteins play a critical role in how learning unfolds in the cerebral cortex. Three of the six proteins identified (BDNF, NR2A, and BCL2) have also been associated with pruning, implying potential for pruning to be linked with learning. Furthermore, BCL2 has been implicated in apoptosis. Learning is fundamentally characterized by a change in brain function. Pruning removes connections between neurons, and apoptosis removes neurons completely; thus, by changing brain structure, pruning and apoptosis potentially modify brain function, potentially playing a critical role in learning. Future research should investigate the potential role of pruning and apoptosis in learning.

### 3.2. Potential Implications: Causality and Correlation

It should be noted that AI results do not themselves imply causality, as these technologies are fundamentally correlation-based. However, it is worthwhile to consider the potential for causal relationships to exist linking our predictor variables (the proteins’ expression levels) and our target variable (learning status). Broadly, AI is a correlation machine, with trained models having identified complex multi-dimensional correlational relationships between predictors (inputs to the AI) and targets (outputs from the AI). As such, independent of AI functionality, actual causal relationships between predictors and targets (or closely related factors) can exist in either direction (predictors causing targets, targets causing predictors) or not at all (only correlations exist in the fundamentals of the data with no underlying causality present). In the context of this study, this implies that if a causal relationship exists, then it might involve protein expression levels (or closely related factors) causing learning, or learning (or closely related factors) causing changes in protein expression levels. In the context of a mouse who was shocked to learn, a process by which they would presumably respond to, or learn from, quite quickly, it seems more likely that protein expression patterns identified are a downstream product of the fundamentals of the learning that has taken place. Perhaps the alternative causal pathway (protein levels lead to learning) could theoretically occur in the case of repetitive conditioned learning, whereby the learner slowly masters a complex task. We know the brain is a highly inter-connected conduit (circuit) for signal transmission [26], with an excess of neurons at birth, estimated at 100 billion [27], leading to fewer neurons in adulthood, with estimates of 86 billion [28] and 67 billion [29], respectively (reviewed in detail in [30]). For cell counts to reduce so dramatically, it seems likely that apoptosis was involved in their removal. In the context of apoptotic processes, pruning could be needed to sever connections between healthy surviving neurons and those undergoing apoptosis, to prevent circuit pathways leading to the absence of a neuron that was removed due to apoptosis. Thus, BCL2′s link to pruning, through aCASP3 expression, which is known to influence synaptic pruning [21,22], is potentially reflective of synaptic pruning of the neurons that previously signaled the now apoptotic cell. Theoretically speaking, when a neuron undergoes apoptosis, or programmed cell death, it is plausible that in terms of neural circuit refinement, the removed apoptotic cell: (1) had outputs that signaled other neurons, and (2) that other neurons formerly signaled the now apoptotic cell. When the outputs of the now apoptotic cell signaled another cell, we should expect the synapse will be removed along with the apoptotic cell as part of the overall cell’s apoptotic processes. However, the synapses from other cells that formerly signaled the now apoptotic cell would no longer serve any function, and so it is plausible that such synapses would undergo synaptic pruning in support of network efficiency, as otherwise the circuit would be left with synapses that signal nothing, and would thus serve no function while contributing to energy consumption. Thus, aCASP3 expression, known to be linked with synaptic pruning [21,22], may be associated with the pruning of synapses that previously signaled the now apoptotic cell. It is noteworthy that in both of the above theoretical scenarios, the pruning that might be occurring that is associated with apoptosis may simply support refinement of the brain’s learning network, such that it remains functionally identical to the same network after the apoptotic cell’s removal in the absence of the associated pruning. Thus, synaptic pruning directly associated with apoptosis may be focused on minor network modifications that result in a functionally identical (or nearly identical) learning network. This does not mean that all forms of pruning are simply modifying the learning network to be functionally identical (other forms of pruning may be very important for supporting learning and thus adapting the brain to new functions) but could imply that such simple examples of pruning, in the form of synaptic pruning, are directly associated with apoptosis through the removal of synapses that formerly signaled the now apoptotic cell.

It is likely that the natural processes of pruning and apoptosis contribute to the removal of tissue and the associated reduction in neurons observed in adulthood [28,29] relative to birth [27]. It is plausible that slow mastery of complex tasks, as part of repetitive conditioned learning, could involve the removal, through pruning and/or apoptosis, of the neural tissue whose function was contributing to errors in the complex task being mastered. In such a situation, it is conceivable that the protein expression levels associated with the pruning of the pathways responsible for errors are an upstream event contributing to the eventual mastery of the complex task learned. However, it is unlikely that this is the case in the present experiment, as rapid learning likely precedes any protein expression level changes, due to the rapid nature of the learning task that the mice were subjected to. That said, it is plausible for rapid learning (such as a mouse being shocked to learn) to lead to pruning and/or apoptosis of neural pathways and tissues that contribute to erroneous learned predictions (in this case, outward behavior that appears to be consistent with a lack of learning).

### 3.3. Discussion of Alternative Analysis

In Section 2.2, we presented the results of an alternative analysis in which the DS mice receiving a placebo saline treatment (no memantine) were placed in the group of mice that had not been shocked to learn, even if they had been. Performance of the learning machines degraded across the validation trials, indicating that these AI models were poorly fitting the dataset as structured, relative to the models from the first method (Section 2.1). The only analytic difference between the two experiments was whether or not the DS mice that did not receive memantine (they received saline), but were shocked to learn, would be placed in the group of interest (those who exhibit learning) or the controls who were not shocked to learn. In this context, a performance drop in AI model predictive accuracy, such as the ones observed in our experiment (see Table 1 vs. Table 3), potentially implies that the group that was moved across analyses (DS mice shocked to learn with placebo) ends up distributed in data space in a manner that overlaps with the distribution of samples from the opposite class. Thus, our findings could imply that the DS mice shocked to learn without treatment likely have protein expression profile characteristics more similar to other mice shocked to learn (DS with treatment, no DS) than to the mice not shocked to learn. Thus, these findings might imply that the DS mice that do not receive treatment and are shocked to learn do have underlying learning processes activating, even if their outward behavior is more consistent with those mice that do not learn. Indeed, Figure 1, which was added during the peer review process, confirms this theory, with the DS group shocked to learn but only receiving saline (no memantine therapy—represented in gray in Figure 1) clearly overlapping in dataspace with the rest of the mice that were shocked to learn (represented in blue in Figure 1). Thus, these findings help motivate future research that includes definitive assessments of whether a mouse successfully learns on an individual basis, and repeating the analysis on such a dataset is part of future work.

### 3.4. Literature Comparison

The first article published on this dataset is focused on the association between protein dynamics and failed and rescued learning [4]. This was a foundational study for this dataset; however, that analysis did not consider the role of machine learning/artificial intelligence in uncovering patterns in the data. The first study to employ AI technology focused on the use of the self-organizing map (SOM), an artificial neural network method for finding groupings of samples (in this case, mice), based on similar feature measurements (in this case, protein expression profiles). The SOM analysis identified 12 features (see their Supplementary Figure S1, in [9]) with potential for discrimination between the mice shocked to learn, and those not [9]. The protein expression biomarkers identified by the SOM method include DYRK1A, pGSK3B, pERK, CaNA, SOD1, pNUMB, ITSN1, IL1B, ubiquitin, PKCA, pPKCAB, and P38 [9]. This provides some consistency with our findings that pERK and SOD1 are highly predictive of learning status. Unfortunately, their approach was focused on unsupervised learning [9], and so we cannot directly compare the predictive accuracy of our models with their study findings, as the equivalent metric is not reported.

Additional existing literature focused on this dataset primarily employs eight-class classification supervised learning frameworks that categorize mice based on genotype (DS or not), treatment (memantine or saline), and behavior (shocked to learn or not) [10,11,12]. In contrast, our study focuses on behavior and introduces a binary classification, thereby enhancing potential biological interpretability. This shift allows targeted identification of proteins whose expression is potentially linked to learning, a critical distinction from prior works that prioritized broad categorization over potential biological insights. All three of the multiclass classification studies focused on this dataset [10,11,12] achieved very high predictive accuracy for creating AI technologies. One of the three previous studies reported 100% accuracy for their leading AI models [11], which are findings similar to our own, as our leading models predict group-wise differences with 100% accuracy using multiple models. Our study distinguishes itself by identifying a small list of proteins whose combined expression levels are predictive of learning, based on a novel redundancy-aware feature selection method. Additionally, in our study design, we have intentionally removed potentially confounding variables (e.g., genotype and treatment) during preprocessing to isolate protein expression potentially associated with learning, as well as to avoid the potential for AI models to be affected by knowledge of either genotype or treatment status.

### 3.5. Machine Learning and Feature Selection

Our leading findings were obtained with logistic regression (lr) and stochastic gradient descent (sgd) using our novel redundancy-aware step-up feature selection method (wrap), achieving 100% predictive accuracy for both technologies (see Table 1). It should be noted that in the absence of employing feature selection (none), predictive accuracy dropped to 97% for sgd and 96.4% for lr. The leading method without feature selection (none) was obtained with the light gradient boosting machine (lgbm), achieving an accuracy of 98%. Thus, these findings imply that our novel redundancy-aware step-up feature selection method has added value by identifying a novel set of six features highly predictive of learning status, achieving predictive performance improvements of 2–3.6% over leading techniques with no feature selection.

### 3.6. Strengths, Limitations, and Future Work

Limitations of this study include that it was performed on a dataset with only 80 total mice, each subjected to a repetitive experiment 15 times, providing only 1200 total samples. An additional limitation of this study was that it was based on a single dataset, as this is the only publicly available dataset of its kind. Future work can verify the findings of our analysis on a larger independent dataset with more samples. Strengths of our analysis include the use of standardized public domain software on a public dataset to support reproducibility, the consideration of a novel feature selection technique, and the identification of a small subset of proteins whose expression is highly predictive of whether a mouse was shocked to learn, potentially furthering our understanding of underlying processes linked with learning in the cerebral cortex. Three of the six highly predictive features have potential links to pruning, and one has been associated with apoptosis. Future work can focus on investigating the potential role of pruning and apoptosis in learning.

## 4. Materials and Methods

### 4.1. Dataset Description

The dataset relied upon in this analysis was collected by a team of researchers trying to determine the effect that medicine, specifically memantine, has on the brain proteins of mice with Down syndrome (DS) [4]. The Ts65Dn mouse model was employed for half of the mice in the study. The Ts65Dn model has been shown to produce mice that have many features associated with DS in humans. They performed context fear conditioning on Ts65Dn and control mice (non-DS) to stimulate learning. Mice were placed in a test chamber and were allowed to explore for a while; then they were shocked, representing the context-shock (CS) group. When placed back into the environment, they would freeze, indicating they had learned to associate the chamber with the shock. Another group was first shocked and then placed in the test chamber; they are the shock-context (SC) group, who were not shocked to learn. The mice were also either injected with the drug memantine or saline as a control. Expression levels for 77 proteins were measured in the cerebral cortex of each mouse. There were a total of 80 mice in the study, binarized on three variables (memantine status, DS status, and learning status), resulting in eight distinct equal-sized classes, outlined in Table 5. Each mouse was subjected to the experiment (being shocked and having their cortical protein expressions measured) 15 times, providing 1200 sets of 77 protein measurements per mouse per experiment [4].

### 4.2. Machine Learning

Data cleaning was performed on this dataset. Protein levels were not normalized in advance; our df-analyze machine learning package conducted its analysis on the native (unprocessed) protein expression levels as input. Preprocessing within df-analyze includes median-based data imputation to handle missing values. Batch effects were handled with mouse-level randomization; this randomizes all of the 15 instances of sets of protein expression levels associated with a single mouse into either the training or testing pools, but does not split them between the two. This was accomplished with df-analyze’s --grouper option applied to the mouse ID field, which was processed [31] to have a unique ID number for each mouse, consistent across each of that mouse’s 15 trials/instances. We performed two main experiments with two unique target variables. In the first experiment, our target was “behavior”, which is a binary classification task whereby the ML predicts whether or not the mouse had been stimulated to learn (learning status). This supports us in identifying specific proteins and their expression levels that are predictive of whether or not the mouse was stimulated to learn. In the second experiment, we focused on predicting whether the mice are *assumed* to have learned. It is noteworthy that in this dataset, we have no ground truth as to whether or not a given mouse actually successfully learned or not, as this was not specifically recorded in the dataset. Instead, we have ground-truth data on whether the mice were stimulated to learn. We also know that the study was constructed, in part, to evaluate the performance of memantine therapy in helping DS mice with their learning capacity. Thus, based on the original study design, a DS mouse with no memantine treatment might have been expected to exhibit substantially impaired learning, whereas a DS mouse with memantine treatment might recover some learning capacity. Mice without DS were expected to be effective learners. As such, for our second experiment, we created a new binary variable, with the target group of interest (binary label 1) consisting of non-DS mice, as well as DS mice with memantine treatment, all subjected to shock learning. The remaining five classes (all mice not subjected to shock learning, as well as DS mice without memantine treatment subjected to shock learning) were assigned to the group-not-of-interest (binary label 0). We have provided code for data processing that was responsible for preparing the data for these experiments [31]. For both experiments outlined, we employed group-based randomization, with each group corresponding to a single mouse’s 15 repeated experiments, thus ensuring that all instances of protein measurements from a single mouse, across all 15 trials, were either assigned to the training or testing groups but not split across both. This helps avoid data contamination and prevents producing biased ML results with potentially overfitted solutions.

For this analysis, we have employed public domain df-analyze software, a tool to simplify complex ML studies, especially for tabular datasets with up to 200,000 samples/rows [32]. It automates many steps, like data type inference, cleaning the dataset by handling missing information through imputation, and model selection. It also automatically splits the data for training, testing, and validation, performs hyperparameter tuning, and thoroughly and fairly evaluates all model and feature selection combinations considered [32]. This public domain ML benchmarking software (version 3.3.0) has been used to study a diverse collection of topics, including schizophrenia [33], chronic kidney disease [34], traffic stop violations [35], and more. For both experiments, an exhaustive comparison of all combinations of supervised learning technologies (LightGBM—lgbm, k-nearest neighbors—knn, logistic regression—lr, random forest—rf, stochastic gradient descent—sgd, as well as a deep learner optimized for tabular data—Gandalf [36], and a baseline model that predicts the majority class—dummy), as well as feature selection methods (filter based association—assoc, filter based prediction—pred, embedded lgbm—embed_lgbm, embedded linear—embed_linear, and an emerging novel redundancy-aware feature selection method [37]—wrap) was performed. Our novel redundancy-aware feature selection method [37] is a new type of step-up feature selection algorithm, which iteratively adds new features to the feature set. Our method is unique in attempting to avoid the addition of features to the feature set whose predictive capacity is redundant relative to the predictive capacity of the feature set already selected. Thus, this technique is biased in favour of finding small sets of features upon which to base predictions, potentially providing valuable feature-specific insights, as well as simpler resultant AI technology whose functionality is easier to explain by virtue of the smaller feature set relied upon. Validation was performed twice for each experiment: once with k-fold validation and once with hold-out validation. We reserved a large 40% of samples for the hold-out set, in order to help produce reproducible findings and avoid producing overfitted solutions. For both validation methods in both experiments, 50 validation runs were performed to help ensure the reliability of results. All reported statistics are averages across the 50 validation runs.

### 4.3. Statistical Analysis

A number of statistical performance metrics are computed to help with the evaluation of ML model performance: accuracy (acc), which assesses the proportion of correct predictions; the area under the receiver operating characteristic curve (auroc), which assesses how well the model can differentiate the group-of-interest from the group-not-of-interest across operating points; balanced accuracy (bal-acc); F1 score (f1); negative predictive value (npv); positive predictive value (ppv); sensitivity (sens); and specificity (spec). Our analysis focuses on the overall accuracy (acc) statistic as a primary metric for model evaluation. In addition to assessing the above standard performance metrics, we also report feature importance scores from feature selection, highlighting the apparent relative importance of each feature measurement (protein expression level) to inform our predictive models. Higher values indicate more predictive importance.

## 5. Conclusions

In this study, we identified cortical molecular biomarkers potentially associated with learning in mice using artificial intelligence (AI). We applied public-domain machine learning software to a public-domain dataset in order to support reproducible findings, with a focus on the development of technologies tasked with predicting whether a given mouse was shocked to learn. Results indicate that it is possible to predict whether a mouse has been shocked to learn highly accurately based only on the following cortical molecular biomarkers: brain-derived neurotrophic factor (BDNF), NR2A—the subunit of N-methyl-D-aspartate receptor, B-cell lymphoma 2 (BCL2), histone H3 acetylation at lysine 18 (H3AcK18), protein kinase R-like endoplasmic reticulum kinase (pERK), and superoxide dismutase 1 (SOD1). These results were obtained with a novel redundancy-aware feature selection method, which outperformed all other feature selection methods, including no feature selection. Five of the six protein expression biomarkers identified have previously been associated with aspects of learning in the literature. Results imply that these six features (BDNF, NR2A, BCL2, H3AcK18, pERK, SOD1) are, in combination with modern artificial intelligence technology, highly predictive of whether or not a mouse has been shocked to learn. Three proteins have been previously associated with pruning, and one with apoptosis, providing motivation to investigate the potential role of pruning and apoptosis in learning as part of future work.

## Figures and Tables

**Table 1:** Results for predicting whether a mouse was shocked to learn. 5-Fold validation on the hold-out set.

Model	Selection	Embed Selector	Acc	AUROC	Bal-Acc	F1	NPV	PPV	Sens	Spec
lr	wrap	none	1.000	1.000	1.000	1.000	1.000	1.000	1.000	1.000
sgd	wrap	none	1.000	1.000	1.000	1.000	1.000	1.000	1.000	1.000
lr	embed_lgbm	lgbm	0.993	1.000	0.993	0.993	1.000	0.984	0.993	0.987
sgd	embed_lgbm	lgbm	0.992	0.996	0.993	0.992	1.000	0.984	0.993	0.987
lgbm	embed_linear	linear	0.983	1.000	0.983	0.983	0.980	0.988	0.983	0.982
lgbm	assoc	none	0.981	1.000	0.981	0.981	0.980	0.984	0.981	0.979
sgd	pred	none	0.981	1.000	0.983	0.981	0.964	1.000	0.983	1.000
lr	embed_linear	linear	0.981	1.000	0.983	0.981	0.972	0.988	0.983	0.990
lgbm	wrap	none	0.980	1.000	0.980	0.979	0.975	0.984	0.980	0.980
lgbm	pred	none	0.980	1.000	0.979	0.979	0.980	0.981	0.979	0.976
lgbm	none	none	0.980	1.000	0.979	0.979	0.980	0.981	0.979	0.976
lgbm	embed_lgbm	lgbm	0.980	1.000	0.979	0.979	0.980	0.981	0.979	0.976
sgd	embed_linear	linear	0.975	1.000	0.978	0.975	0.956	0.991	0.978	0.993
lr	pred	none	0.975	1.000	0.978	0.975	0.955	1.000	0.978	1.000
sgd	assoc	none	0.975	1.000	0.978	0.975	0.961	0.988	0.978	0.990
gandalf	embed_lgbm	lgbm	0.972	0.998	0.969	0.971	0.975	0.976	0.969	0.973
sgd	none	none	0.970	1.000	0.973	0.969	0.952	0.988	0.973	0.990
lr	none	none	0.964	1.000	0.968	0.964	0.941	0.988	0.968	0.990
gandalf	embed_linear	linear	0.958	0.996	0.953	0.955	0.944	0.982	0.953	0.990
rf	embed_linear	linear	0.957	1.000	0.957	0.956	0.976	0.945	0.957	0.934
rf	embed_lgbm	lgbm	0.957	1.000	0.957	0.956	0.973	0.948	0.957	0.937
rf	assoc	none	0.957	1.000	0.957	0.956	0.973	0.948	0.957	0.937
lr	assoc	none	0.956	1.000	0.962	0.956	0.929	0.988	0.962	0.990
rf	none	none	0.954	1.000	0.953	0.952	0.973	0.942	0.953	0.930
rf	pred	none	0.954	0.999	0.953	0.952	0.973	0.942	0.953	0.930
rf	wrap	none	0.954	0.999	0.953	0.952	0.973	0.942	0.953	0.930
knn	embed_lgbm	lgbm	0.953	0.976	0.958	0.946	0.924	0.984	0.958	0.987
gandalf	pred	none	0.948	0.998	0.943	0.927	0.933	0.979	0.943	0.969
knn	wrap	none	0.927	0.938	0.933	0.923	0.929	0.960	0.933	0.950
knn	pred	none	0.924	0.937	0.927	0.915	0.894	0.955	0.927	0.956
knn	embed_linear	linear	0.916	0.950	0.917	0.915	0.881	0.959	0.917	0.956
knn	none	none	0.916	0.950	0.917	0.915	0.881	0.959	0.917	0.956
knn	assoc	none	0.916	0.950	0.917	0.915	0.881	0.959	0.917	0.956
gandalf	none	none	0.889	0.992	0.884	0.883	0.842	0.984	0.884	0.978
gandalf	wrap	none	0.869	0.990	0.879	0.860	0.861	0.964	0.879	0.967
gandalf	assoc	none	0.830	0.954	0.834	0.826	0.788	0.924	0.834	0.921
dummy	embed_linear	linear	0.443	0.500	0.500	0.307	0.429	0.452	0.500	0.400
dummy	none	none	0.443	0.500	0.500	0.307	0.429	0.452	0.500	0.400
dummy	assoc	none	0.443	0.500	0.500	0.307	0.429	0.452	0.500	0.400
dummy	embed_lgbm	lgbm	0.443	0.500	0.500	0.307	0.429	0.452	0.500	0.400
dummy	pred	none	0.443	0.500	0.500	0.307	0.429	0.452	0.500	0.400
dummy	wrap	none	0.443	0.500	0.500	0.307	0.429	0.452	0.500	0.400

Acc = Accuracy, Bal-Acc = Balanced Accuracy, NPV = Negative Predictive Value, PPV = Positive Predictive Value, Sens = Sensitivity, Spec = Specificity.

**Table 2:** Redundancy-aware feature selection report predicting shocked-to-learn status.

Feature	Score
SOD1_N_	0.944
pERK_N_	0.994
BDNF_N_NAN_	0.994
NR2A_N_	0.964
H3AcK18_N_NAN_	0.983
BCL2_N_NAN_	0.961

Values rounded to 3 decimal places for clarity.

**Table 3:** Results for the alternative approach to detecting potentially learning linked proteins. 5-fold validation on the hold-out set.

Model	Selection	Embed Selector	Acc	AUROC	Bal-Acc	F1	NPV	PPV	Sens	Spec
sgd	embed_lgbm	lgbm	0.877	0.893	0.876	0.873	0.850	0.917	0.876	0.891
rf	none	none	0.876	0.890	0.889	0.875	0.812	0.959	0.889	0.964
rf	pred	none	0.874	0.893	0.887	0.873	0.811	0.955	0.887	0.960
rf	embed_linear	linear	0.874	0.893	0.887	0.873	0.811	0.955	0.887	0.960
knn	embed_lgbm	lgbm	0.873	0.889	0.867	0.869	0.853	0.889	0.867	0.840
rf	assoc	none	0.872	0.875	0.883	0.871	0.811	0.952	0.883	0.953
rf	wrap	none	0.872	0.908	0.884	0.870	0.803	0.971	0.884	0.969
lr	embed_lgbm	lgbm	0.860	0.917	0.847	0.853	0.872	0.863	0.847	0.796
gandalf	none	none	0.825	0.903	0.771	0.773	0.914	0.810	0.771	0.596
lgbm	wrap	none	0.823	0.900	0.807	0.805	0.799	0.884	0.807	0.804
gandalf	pred	none	0.819	0.918	0.792	0.799	0.888	0.801	0.792	0.660
gandalf	embed_lgbm	lgbm	0.817	0.926	0.782	0.765	0.913	0.814	0.782	0.618
lgbm	embed_lgbm	lgbm	0.815	0.920	0.797	0.799	0.824	0.858	0.797	0.758
rf	embed_lgbm	lgbm	0.809	0.859	0.797	0.797	0.815	0.852	0.797	0.764
lr	none	none	0.808	0.894	0.794	0.798	0.778	0.850	0.794	0.762
lr	embed_linear	linear	0.806	0.891	0.792	0.796	0.778	0.847	0.792	0.758
lgbm	pred	none	0.806	0.878	0.801	0.800	0.769	0.850	0.801	0.789
knn	wrap	none	0.805	0.867	0.775	0.789	0.840	0.800	0.775	0.667
sgd	embed_linear	linear	0.803	0.821	0.800	0.798	0.775	0.844	0.800	0.787
lr	assoc	none	0.800	0.891	0.784	0.789	0.777	0.842	0.784	0.749
sgd	wrap	none	0.799	0.899	0.768	0.778	0.808	0.807	0.768	0.669
lr	wrap	none	0.799	0.904	0.771	0.781	0.808	0.805	0.771	0.673
sgd	none	none	0.799	0.898	0.787	0.790	0.778	0.839	0.787	0.760
sgd	pred	none	0.798	0.879	0.790	0.791	0.779	0.820	0.790	0.738
lgbm	assoc	none	0.797	0.878	0.784	0.787	0.784	0.839	0.784	0.758
sgd	assoc	none	0.796	0.808	0.782	0.786	0.775	0.835	0.782	0.751
gandalf	assoc	none	0.792	0.886	0.744	0.748	0.833	0.781	0.744	0.547
lr	pred	none	0.791	0.883	0.786	0.784	0.782	0.818	0.786	0.738
lgbm	embed_linear	linear	0.788	0.889	0.773	0.776	0.781	0.831	0.773	0.736
lgbm	none	none	0.783	0.888	0.764	0.768	0.790	0.818	0.764	0.709
knn	pred	none	0.746	0.785	0.733	0.729	0.741	0.774	0.733	0.649
knn	embed_linear	linear	0.745	0.800	0.722	0.730	0.732	0.764	0.722	0.629
knn	assoc	none	0.745	0.800	0.722	0.730	0.732	0.764	0.722	0.629
knn	none	none	0.745	0.800	0.722	0.730	0.732	0.764	0.722	0.629
gandalf	wrap	none	0.730	0.865	0.707	0.687	0.808	0.784	0.707	0.629
dummy	embed_linear	linear	0.611	0.500	0.500	0.378	–	0.611	0.500	0.000
dummy	pred	none	0.611	0.500	0.500	0.378	–	0.611	0.500	0.000
dummy	none	none	0.611	0.500	0.500	0.378	–	0.611	0.500	0.000
dummy	assoc	none	0.611	0.500	0.500	0.378	–	0.611	0.500	0.000
dummy	embed_lgbm	lgbm	0.611	0.500	0.500	0.378	–	0.611	0.500	0.000
dummy	wrap	none	0.611	0.500	0.500	0.378	–	0.611	0.500	0.000
gandalf	embed_linear	linear	0.589	0.696	0.539	0.435	0.358	0.638	0.539	0.311

Acc = Accuracy, Bal-Acc = Balanced Accuracy, NPV = Negative Predictive Value, PPV = Positive Predictive Value, Sens = Sensitivity, Spec = Specificity.

**Table 4:** Redundancy-aware feature selection report—alternative analysis.

Feature	Score
CaNAN	0.720
SOD1N	0.893
pP70S6N	0.906
BADN_NAN	0.912
UbiquitinN	0.916
H3AcK18N	0.918
pRSKN	0.914
pPKCABN	0.901
NR2AN	0.905
pCASP9N	0.903
GSK3BN	0.897
pCAMKIIN	0.862

Values rounded to 3 decimal places for clarity.

**Table 5:** Dataset description.

Measurement	Data	Description
Genotype	c	Control mouse
	t	Trisomy mouse (Ts65Dn model)
Treatment Type	m	Mouse injected with Memantine
	s	Mouse injected with saline (control)
Behavior	CS	Context-shock: Mice explored test chamber before shock
	SC	Shock-context: Mice received shock before exploration
Class (Target)	c-CS-s	Control mouse: Context-shock conditioning + saline
	c-CS-m	Control mouse: Context-shock conditioning + memantine
	c-SC-s	Control mouse: Shock-context conditioning + saline
	c-SC-m	Control mouse: Shock-context conditioning + memantine
	t-CS-s	Ts65Dn mouse: Context-shock conditioning + saline
	t-CS-m	Ts65Dn mouse: Context-shock conditioning + memantine
	t-SC-s	Ts65Dn mouse: Shock-context conditioning + saline
	t-SC-m	Ts65Dn mouse: Shock-context conditioning + memantine

CS = Context-Shock, SC = Shock-Context.

## Data Availability

The dataset used in this study is publicly available and can be accessed from OpenML at https://www.openml.org/search?type=data&sort=runs&status=active&qualities.NumberOfInstances=between_1000_10000&order=desc&id=40966, accessed on 30 September 2024. No new data were created or collected specifically for this study. Since this was a retrospective analysis of public-domain data, no institutional review board approval was necessary for conducting this study.

## Abbreviations

The following abbreviations are used in this manuscript:

acc	accuracy
AI	artificial intelligence
assoc	filter-based association FS
auroc	area under the receiver operating characteristic curve
bal-acc	balanced accuracy
BCL2	B-cell lymphoma 2
BDNF	brain-derived neurotrophic factor
DS	Down syndrome
embed_lgbm	embedded lgbm FS
embed_linear	embedded linear FS
f1	F1 score
FS	feature selection
H3AcK18	histone H3 acetylation at lysine 18
KNN	k-nearest neighbor
lgbm	Light Gradient-Boosting Machine
lr	logistic regression
ML	Machine Learning
NDMA	N-methyl-D-aspartate receptor
npv	Negative Predictive Value
NR2A	the subunit of the NDMA receptor
pERK	protein kinase R-like endoplasmic reticulum kinase
ppv	positive predictive value
pred	filter-based prediction FS
rf	random forest
S2L	shocked to learn
sens	sensitivity
sgd	stochastic gradient descent
SOD1	superoxide dismutase 1
spec	specificity
SOM	self-organizing map
wrap	wrapper-based redundancy aware FS
